# An invertebrate infection model for evaluating anti-fungal agents against dermatophytosis

**DOI:** 10.1038/s41598-017-12523-z

**Published:** 2017-09-25

**Authors:** Masaki Ishii, Yasuhiko Matsumoto, Tsuyoshi Yamada, Shigeru Abe, Kazuhisa Sekimizu

**Affiliations:** 1 Genome Pharmaceuticals Institute Co. Ltd., 102 Next Building, 3-24-17 Hongo, Bunkyo-ku, Tokyo 113-0033 Japan; 2 Teikyo University Institute of Medical Mycology, 359 Otsuka, Hachioji, Tokyo 192-0395 Japan

## Abstract

Animal models of pathogenic infection are needed to evaluate candidate compounds for the development of anti-infectious drugs. Dermatophytes are pathogenic fungi that cause several infectious diseases. We established a silkworm dermatophyte infection model to evaluate anti-fungal drugs. Injection of conidia of the dermatophyte *Arthroderma vanbreuseghemii* into silkworms was lethal. *A. vanbreuseghemii* conidia germinated in liquid culture were more potent against silkworms than non-germinated conidia. Germinated conidia of other dermatophytes, *Arthroderma benhamiae*, *Trichophyton rubrum*, and *Microsporum canis*, also killed silkworms. Injection of heat-treated germinated *A. vanbreuseghemii* conidia did not kill silkworms, suggesting that only viable fungi are virulent. Injecting terbinafine or itraconazole, oral drugs used clinically to treat dermatophytosis, into the silkworm midgut had therapeutic effects against infection with germinated *A. vanbreuseghemii* conidia. When silkworms were injected with *A. vanbreuseghemii* expressing enhanced green fluorescent protein (eGFP), mycelial growth of the fungus was observed in the fat body and midgut. Injection of terbinafine into the silkworm midgut, which corresponds to oral administration in humans, inhibited the growth of *A. vanbreuseghemii* expressing eGFP in the fat body. These findings suggest that the silkworm infection model with eGFP-expressing dermatophytes is useful for evaluating the therapeutic activity of orally administered anti-fungal agents against dermatophytes.

## Introduction

Pathogenic fungi cause several infectious diseases, such as superficial cutaneous fungal infection and serious deep infection^1^. Superficial cutaneous fungal infection affects a quarter of the human population worldwide, and the prevalence is increasing^2^. Dermatophytes, pathogenic fungi, can cause serious deep infection in immunocompromised individuals^3,4^. Anti-fungal drugs against dermatophytosis in humans are administered orally or applied topically. The complete cure rate of dermatophytosis of the toenail by treatment with the topical medicine efinaconazol, however, is lower than 17.8–22.2%^5^. Furthermore, oral medicines used clinically against fungal diseases interact with many drugs and may cause serious side effects^6,7^. Therefore, the development of novel, effective anti-fungal drugs against dermatophytosis with fewer side effects is needed.

Most chemicals that exhibit antimicrobial activity *in vitro* do not have therapeutic effects in animal models because of their *in vivo* pharmacokinetics and toxicity. Therefore, evaluation of therapeutic effects in animal models is necessary for the development of clinically effective anti-fungal agents. Mammalian animals such as the guinea pig are often used as fungal skin infection models^8^. The high costs and ethical issues associated with using a large number of mammalian animals to screen for novel therapeutic agents against dermatophytosis, however, are prohibitive.

The silkworm is a useful animal model to evaluate the therapeutic effects of anti-bacterial and anti-viral drugs before testing the compounds in mammalian animals^9,10^. Silkworms are much less expensive to rear and maintain than mammalian animals, and their experimental use partly avoids the ethical issues. The silkworm size is relatively large and thus sufficient for different compound administration routes, such as intra-hemolymph and intra-midgut injection using a tuberculin syringe^11^. Intra-midgut injection in silkworms corresponds to oral administration in mammals. Amphotericin B, which is not orally available for human patients, also does not exhibit therapeutic effects in silkworms when administered into the intestine^12^. The median effective dose (ED_50_) of antibiotics in silkworms is consistent with that in mammals^13^. Furthermore, the pharmacokinetic parameters of antibiotics, such as the half-life in blood and protein binding capacities are also consistent between silkworms and mammals^14,15^. The LD_50_ value, the amount of reagents required to kill 50% of animals, of chemicals against silkworms also correlates well with that in mammals^16,17^, and therefore quantitative evaluation of both the toxic and therapeutic effects of candidate compounds can be simultaneously performed silkworms. By using the silkworm infection model as a second screening tool after an initial *in vitro* screening, we recently discovered a novel antibiotic from soil bacteria, lysocin E, that is therapeutically effective in a mouse model of systemic *Staphylococcus aureus* infection^10^.

Silkworm systemic infection models of five fungal species, *Candida albicans*, *C. tropicalis*, *C. glabrata*, *Cryptococcus neoformans*, and *Aspergillus fumigatus*, have been established^13,18–21^. These silkworm infection models can be used to quantitatively evaluate the therapeutic efficacy of anti-fungals. Using a silkworm infection model with the filamentous fungus *Aspergillus fumigatus*, we discovered ASP2397, a compound that has therapeutic effects in a mouse infection model^20^. This finding suggests that silkworms are useful for identifying novel anti-fungal drugs. To evaluate anti-fungal drugs against dermatophytes, we aimed to establish a novel silkworm infection model.

For evaluation of the therapeutic efficacy of drugs against dermatophytes, it is important to be able to quantitatively assess dermatophyte growth in animals. Dermatophytes grow as filamentous structures called hyphae, collectively referred to as a mycelium. The mycelial growth of dermatophytes contributes to disease progression^22–24^. Quantitative evaluation of mycelial growth in animals is difficult, however, because a single hypha may comprise numerous cells^25^. To overcome this problem, we aimed to establish a system to quantitatively evaluate dermatophyte growth *in vivo*. Fluorescence imaging is a simple and effective method of quantitatively evaluating cell growth and mobility *in vivo* ^26^. Green fluorescent protein (GFP) is a reported protein that is stable *in vivo* ^27^. Dermatophytes expressing enhanced GFP (eGFP) were established^28^. We hypothesized that mycelial growth could be evaluated based on detection of the fluorescence of eGFP-expressing dermatophytes in animals. Here we describe a silkworm infection model with dermatophytes expressing eGFP, and demonstrate that the infection model is useful for evaluating the therapeutic effects of terbinafine, a drug used clinically to treat dermatophyte infection.

## Results

### Killing of silkworms by injection of dermatophyte conidia

**Figure 1:**
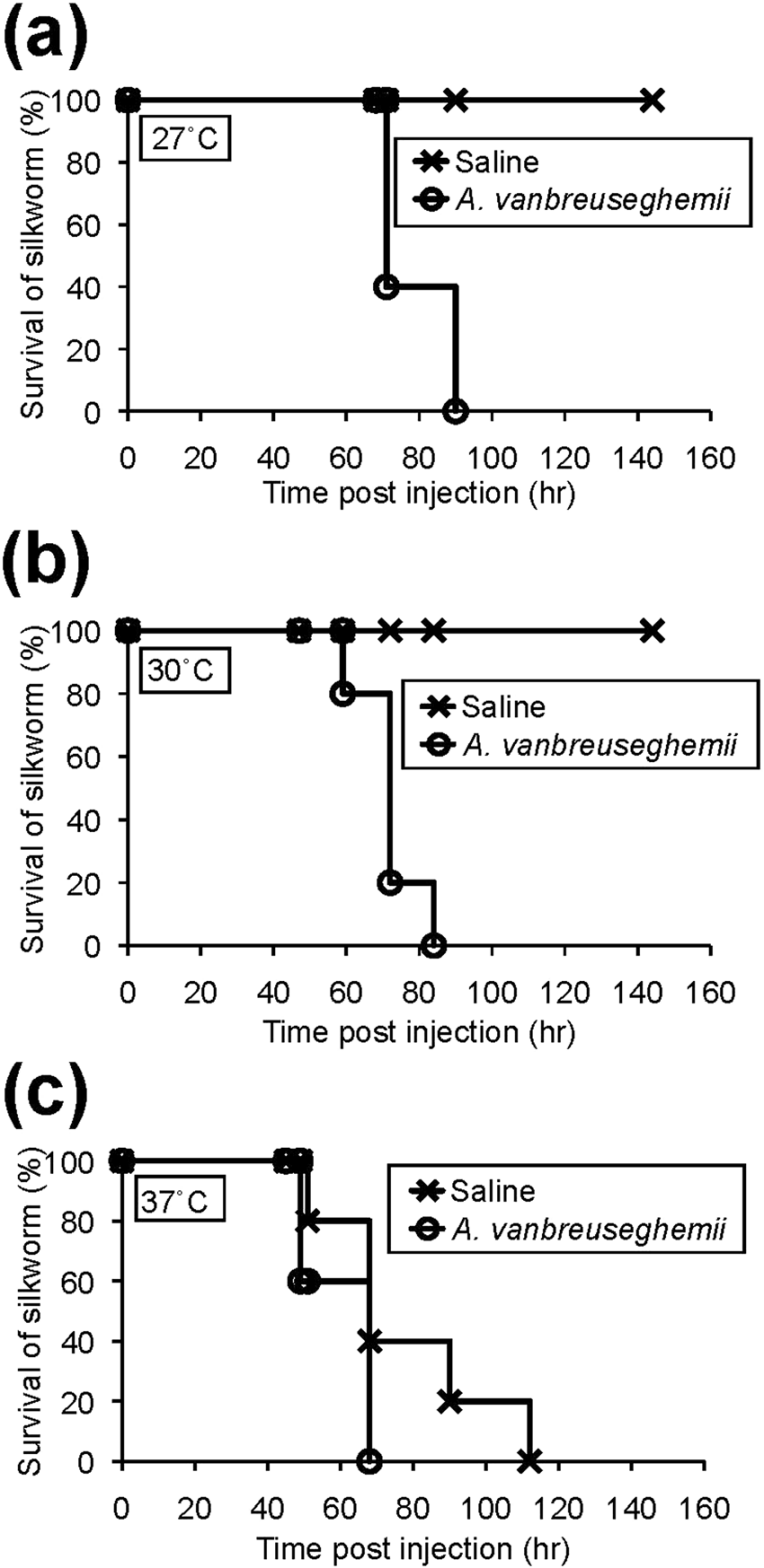
Effect of temperature on the pathogenicity of the *Arthroderma vanbreuseghemii* TIMM2789 dermatophytes in silkworms. Conidia (4 × 10^6^) of *A. vanbreuseghemii* TIMM2789 dermatophytes were injected into the silkworm hemolymph. Survival of the animals at 27 °C (**a**), 30 °C (**b**), and 37 °C (**c**) was monitored. n = 5/group.

Previous studies of silkworm infection models of *C. albicans*, *C. glabrata*, *C. tropicalis*, *C. neoformans*, and *Aspergillus fumigatus* demonstrated that rearing temperatures after fungal injection profoundly affect the results^29^. The appropriate temperatures for infectious experiments are 27 °C for *C. albicans* and *C. tropicalis*; 30 °C for *A. fumigatus*; and 37 °C for *C. glabrata* and *C. neoformans*. Therefore, to establish the dermatophyte infection model, we first performed experiments at different temperatures. At 27 °C and 30 °C, all silkworms injected with the conidia of *Arthroderma vanbreuseghemii* dermatophytes died within 100 h, whereas all silkworms injected with saline were alive (Fig. 1a,b). On the other hand, at 37 °C, all silkworms injected with saline died, resulting in a small difference in the survival period between injection with saline and injection with dermatophytes (Fig. 1c). Subsequent infectious experiments with dermatophyte conidia were conducted at 30 °C, the temperature at which silkworm death was dependent on dermatophyte injection.

**Figure 2:**
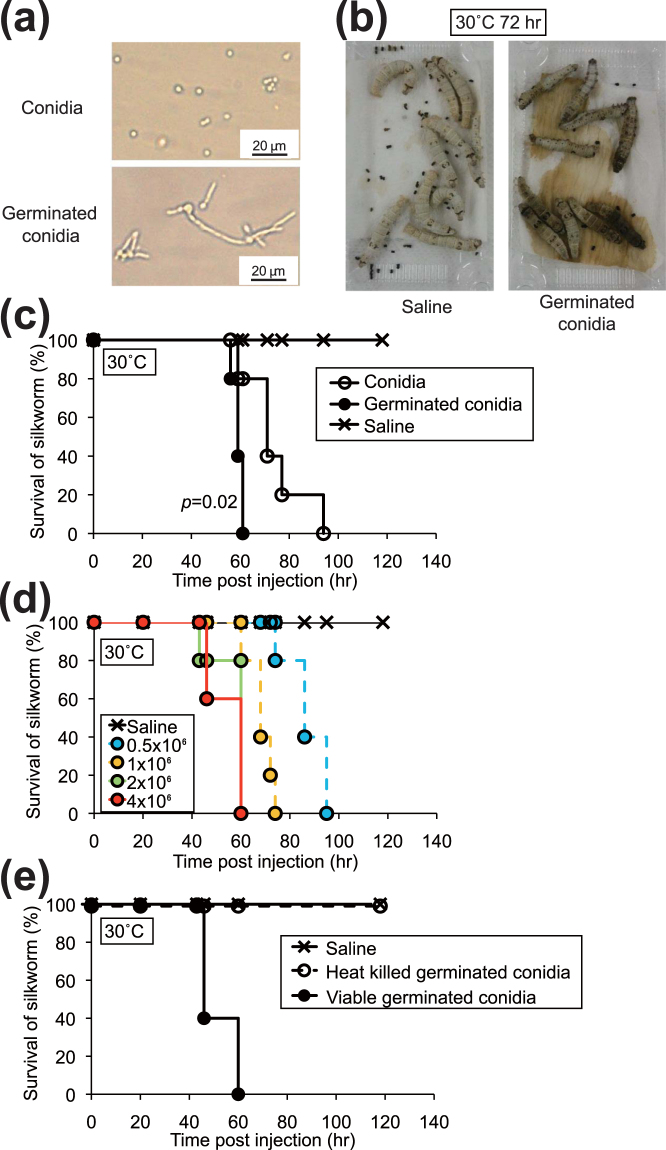
Infection of silkworms with germinated conidia of *A. vanbreuseghemii* TIMM2789 dermatophytes. (**a**) Microscopic observation of conidia of *A. vanbreuseghemii* TIMM2789 dermatophytes (upper panel) and germinated conidia cultured at 28 °C for 26 h (lower panel). (**b**) Silkworms reared at 30 °C for 72 h after injection of 4 × 10^6^ of germinated conidia (right). Saline control (left). n = 10/group. (**c**) Conidia of *A. vanbreuseghemii* TIMM2789 dermatophytes were cultured at 28 °C for 26 h (germinated conidia). Conidia (4 × 10^6^) or germinated conidia (4 × 10^6^) were injected into the silkworm hemolymph, and the silkworms were reared at 30 °C. Survival of the animals was monitored. n = 5/group. (**d**) Conidia of *A. vanbreuseghemii* TIMM2789 dermatophytes were cultured at 28 °C for 26 h. Dermatophytes (0.5–4 × 10^6^) were injected into the silkworms, and the silkworms were reared at 30 °C. Survival of the animals was monitored. n = 5/group. (**e**) Conidia of *A. vanbreuseghemii* TIMM2789 dermatophytes were cultured at 28 °C for 24 h. Germinated conidia (8 × 10^6^) or samples autoclaved at 121 °C for 15 min (heat-killed germinated conidia) were injected into silkworms, and silkworms were reared at 30 °C. Survival of the animals was monitored. n = 5/group.

Dermatophyte conidia germinate in rich nutrient media, and form hyphae. Hyphal growth of dermatophytes contributes to the progression of infectious disease^22–24^. We tested whether conidia germinating in liquid media were more lethal to silkworms than those without germination. Germinated conidia were formed 1 day after incubation in liquid media (Fig. 2a) and the germinated conidia killed silkworms (Fig. 2b). Silkworms were killed more quickly by injection of germinated conidia than by injection of non-germinated conidia (Fig. 2c). The lethality of germinated conidia in silkworms was dose-dependent, ranging from 0 to 4 × 10^6^ cells/larva (Fig. 2d). On the other hand, heat treatment of germinated conidia at 121 °C for 15 min abolished their lethal effects in silkworms (Fig. 2e).

**Figure 3:**
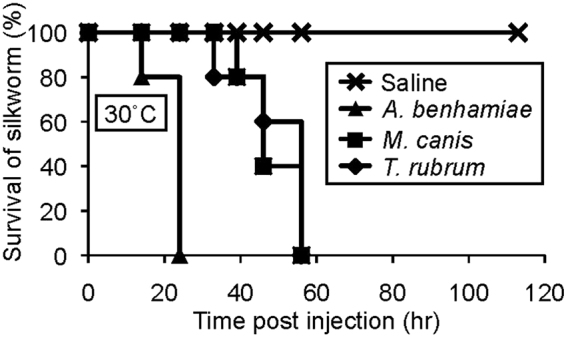
Pathogenicity of germinated conidia of *Trichophyton rubrum*, *A. benhamiae*, and *Microsporum canis* dermatophytes to silkworms. Conidia of *Trichophyton rubrum*, *A. benhamiae*, and *Microsporum canis* dermatophytes were cultured at 28 °C for 21 h. Conidia (2 × 10^6^) were injected into silkworms, and the silkworms were reared at 30 °C. Survival of the animals was monitored. n = 5/group.

Next, we examined the virulence of other dermatophytes in silkworms. Several fungal species cause dermatophytosis in humans^30^. Among them, *Trichophyton rubrum* is clinically isolated with the highest frequency. *A. benhamiae* and *Microsporum canis* also cause dermatophytosis. Silkworms were also killed by injection into the hemolymph of the germinated conidia of *T. rubrum*, *A. benhamiae*, and *M. canis* (Fig. 3, Table 1). These findings suggest that the silkworm infection model is useful for quantitatively evaluating dermatophyte virulence.

**Table 1:** LD_50_ values of dermatophytes against silkworms.

Strains	LD_50_ ± SD (×10^5^ germinated spore/silkworm)
*A. vanbreuseghemii* TIMM2789	9 ± 1
*A. benhamiae* IHEM20161	0.6 ± 0.1
*M. canis* 20080	2 ± 0.6
*T. rubrum* CBS118892	5 ± 2

Germinated conidia were injected into the silkworm hemolymph, and the silkworms were reared at 30 °C. Survival of the animals was monitored after 72 hours (n = 5/group). LD_50_ values of dermatophytes were determined from three independent experiments.

### Evaluation of therapeutic effects of anti-fungal drugs using the silkworm infection model

**Figure 4:**
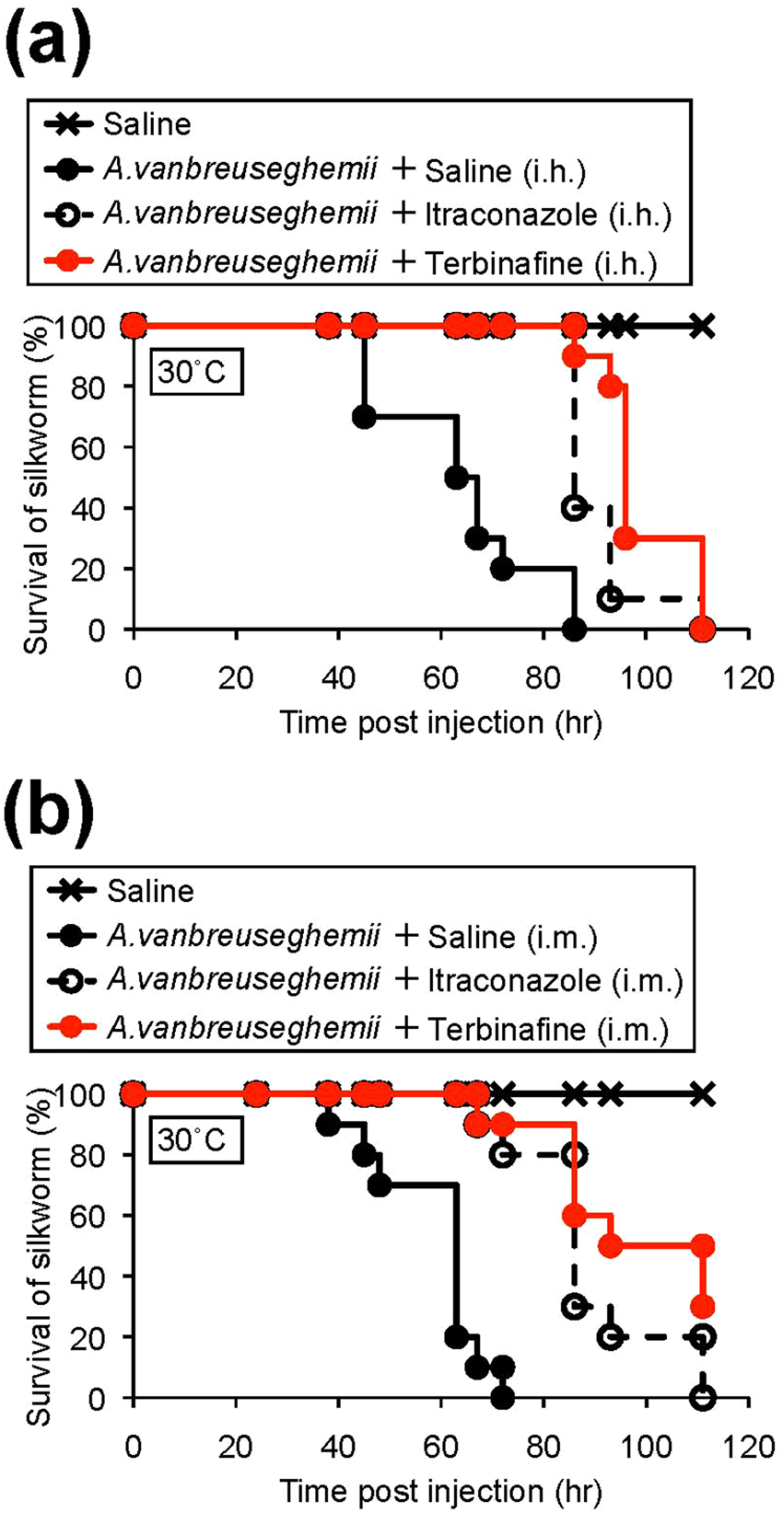
Therapeutic effects of terbinafine and itraconazole in silkworms infected with *A. vanbreuseghemii* TIMM2789 dermatophytes. Conidia of *A. vanbreuseghemii* TIMM2789 dermatophytes were cultured at 28 °C for 24 h. Cultured conidia (4 × 10^6^) were injected into the silkworm hemolymph, followed by injection of 20 μg of terbinafine or 50 μg of itraconazole into hemolymph (i.h.: intra-hemolymph injection) or midgut (i.m.: intra-midgut injection). The silkworms were reared at 30 °C, and survival of the animals was monitored. n = 10/group.

We next examined whether anti-fungal drugs can be quantitatively evaluated using the silkworm dermatophyte-infection model. Oral administration of terbinafine or itraconazole is effective in human patients^31,32^. Intra-hemolymph injection of terbinafine or itraconazole extended silkworm life compared with silkworms injected with *A. vanbreuseghemii* alone (Fig. 4a). Injection of terbinafine or itraconazole into the midgut also had therapeutic effects against *A. vanbreuseghemii* infection (Fig. 4b). The ED_50_ values of terbinafine and itraconazole following intra-midgut injection were 11 ± 3 and 21 ± 3 μg/kg, respectively, against infection with *A. vanbreuseghemii* (Table 2). These results suggest that the therapeutic effects of anti-fungal drugs can be quantitatively evaluated using the silkworm dermatophyte infection model.

**Table 2:** MIC and ED_50_ values of terbinafine and itraconazole against *A. vanbreuseghemii* TIMM2789.

	MIC_80_ (μg·ml^−1^)	ED_50_ of i.h. (mg·kg^−1^ of larva)	ED_50_ of i.m. (mg·kg^−1^ of larva)
Terbinafine	0.013	5.3 ± 2.9	11 ± 3
Itraconazole	0.060	17 ± 6	21 ± 3

Germinated conidia were injected into the silkworm hemolymph, followed by injection of terbinafine or itraconazole into the hemolymph (i.h.) or midgut (i.m.). Silkworms were reared at 30 °C. Survival of the animals was monitored after 72 hours (n = 3/dose). ED_50_ values of anti-fungal agents were determined from three independent experiments.

### Fluorescence imaging analysis of dermatophyte infection

**Figure 5:**
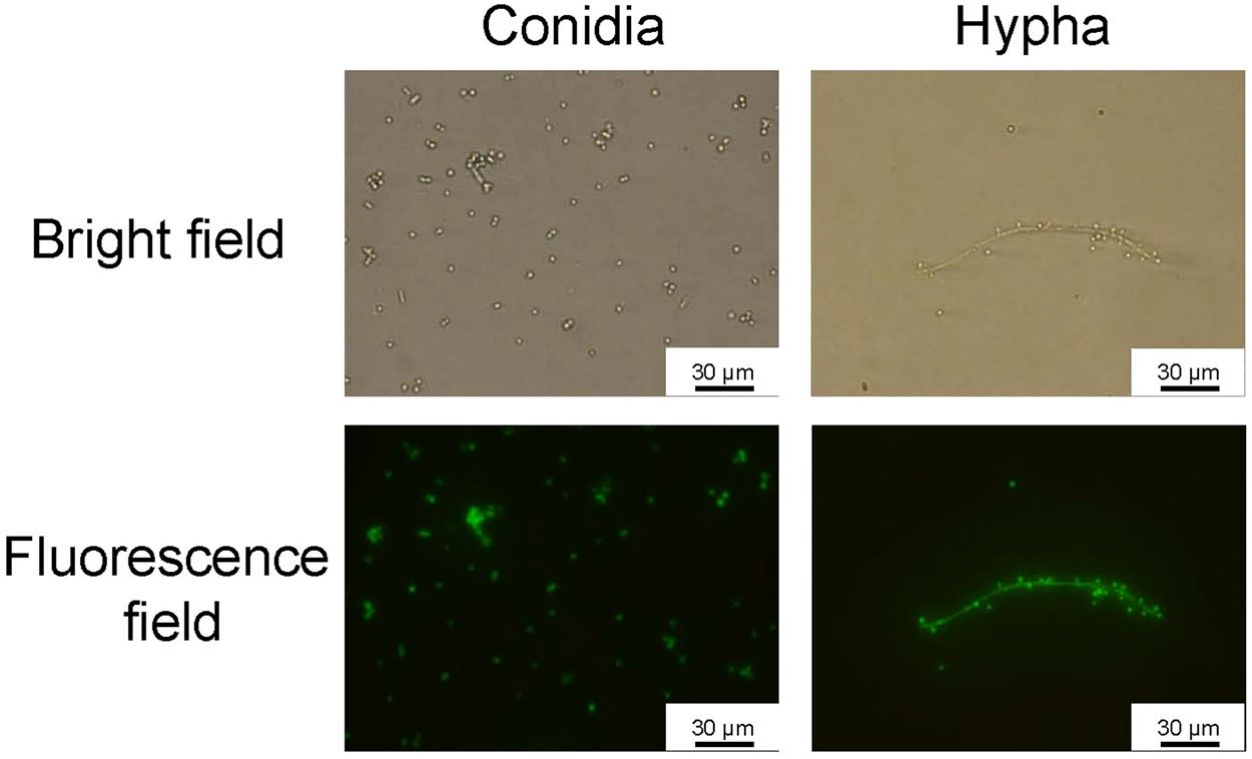
Microscopic observation of *A. vanbreuseghemii* dermatophytes expressing eGFP. Left panels: Conidia of *A. vanbreuseghemii* expressing eGFP. Right panels: Hyphae of *A. vanbreuseghemii* expressing eGFP. Upper panels: bright field. Lower panels: fluorescence field.

**Figure 6:**
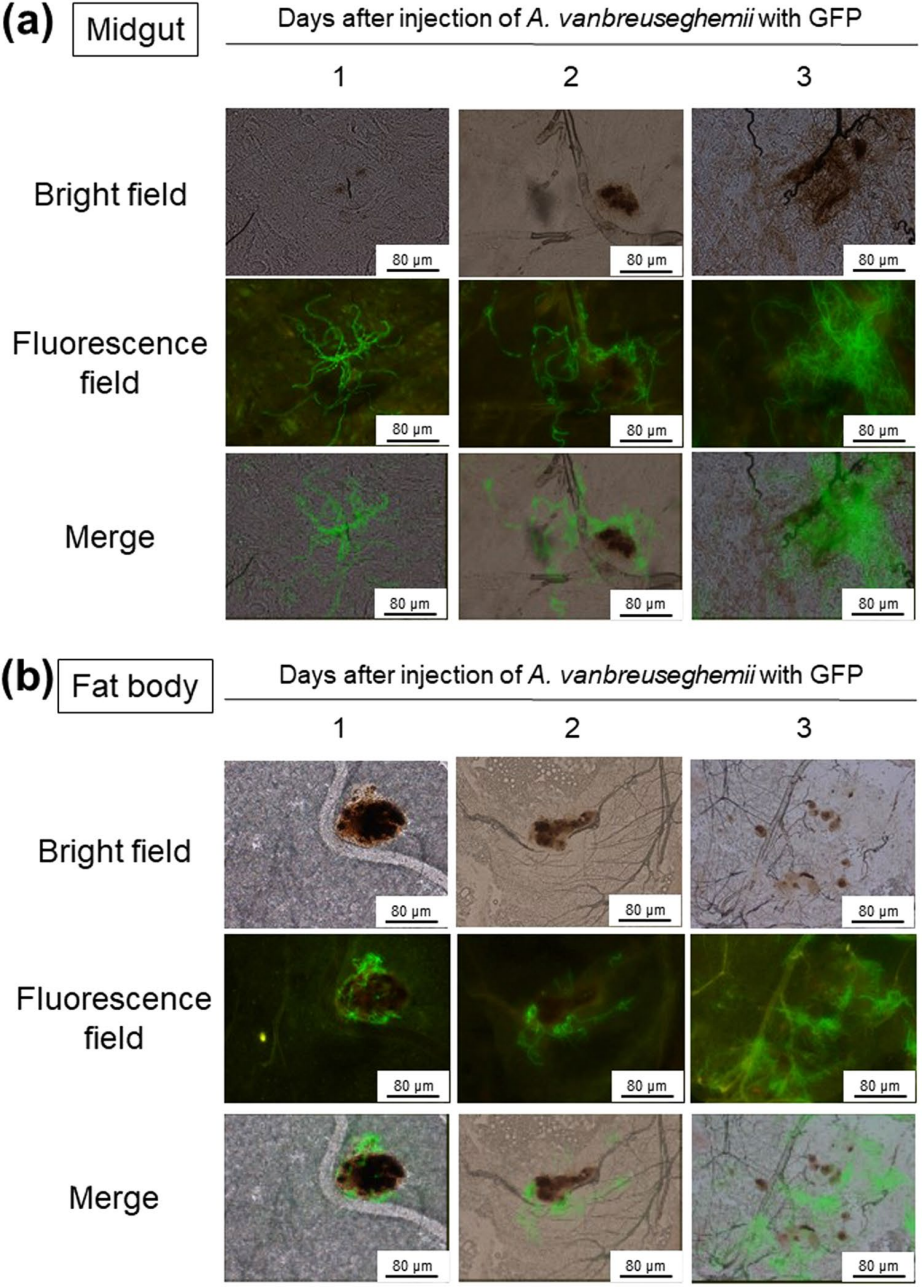
Fluorescence imaging of tissues of silkworms infected with *A. vanbreuseghemii* dermatophytes expressing eGFP. Conidia (5 × 10^6^) of *A. vanbreuseghemii* expressing eGFP were injected into silkworms. The silkworms were reared at 30 °C for 1, 2, and 3 days. Silkworms were dissected and the midgut (**a**) or fat body (**b**) was isolated. Small pieces of fat body and midgut were placed on glass slides and pressed flat by cover slips. The tissues were observed under an inverted microscope (in brightfield, under excitation light, and merged images).

**Figure 7:**
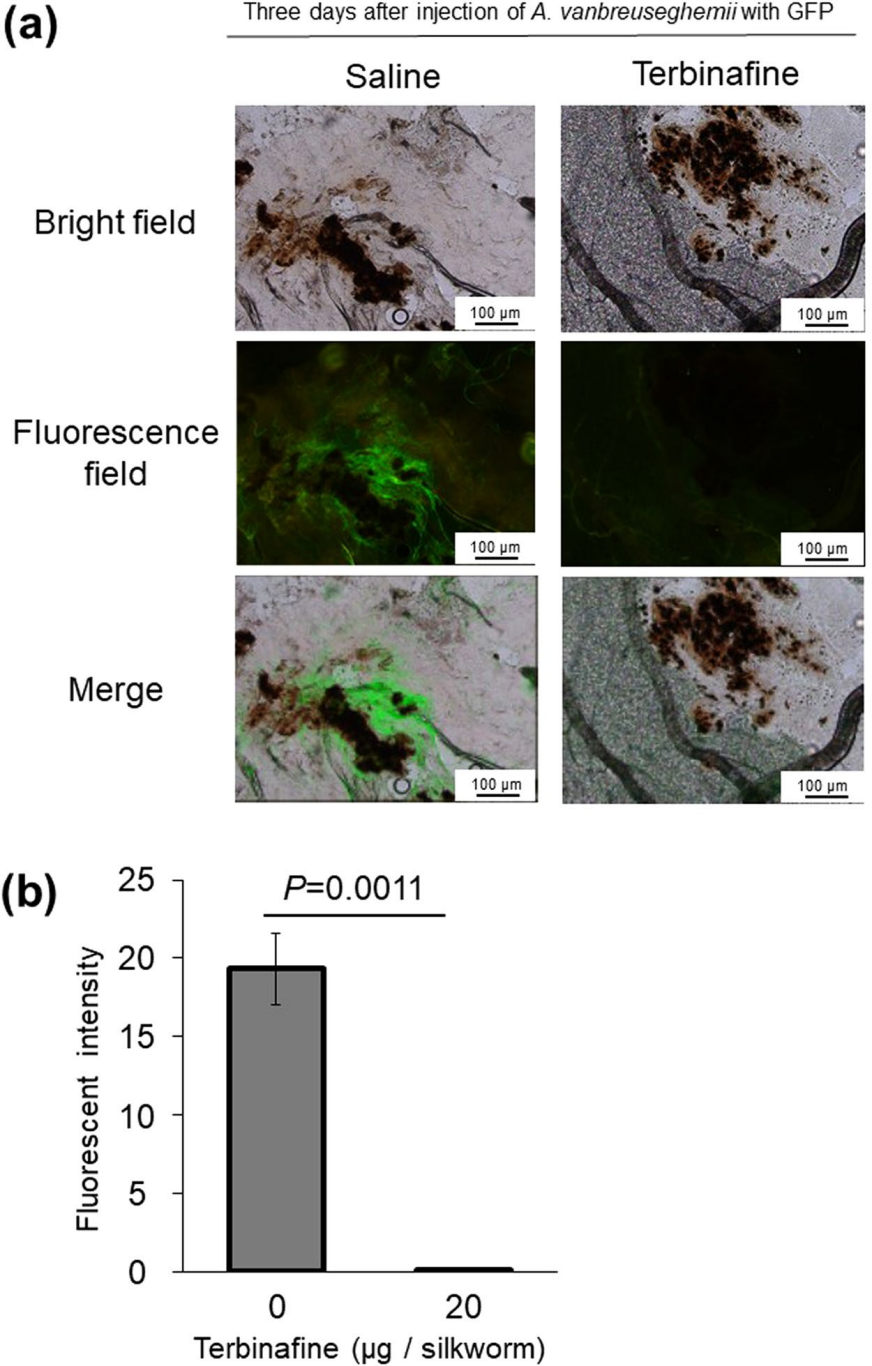
Therapeutic effects of terbinafine on silkworms infected with *A. vanbreuseghemii* dermatophytes expressing eGFP. Conidia (5 × 10^6^) of *A. vanbreuseghemii* dermatophytes expressing eGFP were injected into silkworms, followed by injection of terbinafine (20 μg) into the silkworm midgut. The silkworms were reared at 30 °C for 3 days. (**a**) Small pieces of fat body were placed on glass slides and pressed flat by cover slips. The samples were observed under a microscope (in brightfield, under excitation light, and merged images). (**b**) The fat body tissue was homogenized by sonication and the centrifuged pellet was suspended in water. The suspended sample (0.5 μl) was dropped on a microscope slide and dried. The samples were observed under a fluorescence microscope. After obtaining an image of the homogenized tissue, the fluorescence intensity of the sample area was determined. Florescence intensity/μg ∙ tissue protein is presented. n = 3/group.

To observe the growth of dermatophytes in silkworms, we established a fluorescence imaging system with dermatophytes expressing eGFP. We confirmed the fluorescence of the conidia and hypha of dermatophytes expressing eGFP (Fig. 5). After injecting the conidia of dermatophytes expressing eGFP, mycelial growth was observed in the silkworm midgut and fat body (Fig. 6). Melanization, an insect immune response, was observed as brown spots in isolated silkworm tissues infected with dermatophytes and as brown filaments in the trachea, a silkworm respiratory organ. Injection of terbinafine inhibited the mycelial growth of dermatophytes expressing eGFP in the fat body at day 3 (Fig. 7a). Growth of the mycelia in the fat body was quantified by measuring fluorescence per weight of the organ and mycelial growth was inhibited in the terbinafine-injected group (Fig. 7b).

## Discussion

The present study demonstrated that injection of dermatophytes into silkworm hemolymph killed silkworms. By injecting *A. vanbreuseghemii* expressing eGFP into the silkworms, we were able to observe that terbinafine inhibited the mycelial growth of the dermatophyte. These findings suggest that the silkworm dermatophyte infection model is useful for evaluating the therapeutic effects of anti-fungal agents.

Although a dermatophyte infection model using mammals has been proposed, dermatophytes did not have lethal effects in that model. Injection of dermatophytes kills the larvae of *Galleria mellonella*, a lepidopteran insect like silkworm^33^. In the *Galleria mellonella* system, heat-killed dermatophytes were still lethal, indicating that the virulence of the dermatophytes is not due to the growth of the pathogens. In such a system, it is difficult to evaluate the therapeutic effects of compounds that exhibited anti-fungal activity *in vitro*. We demonstrated that injection of dermatophytes into silkworms is only lethal if the fungi are viable. Furthermore, anti-fungal drugs inhibited the growth and lethality of the dermatophyte in the silkworms. These findings indicate that mycelial growth is required for the dermatophyte to be pathogenic in silkworms. This is an advantage of the silkworm system compared with the *G. mellonella* system for evaluating the therapeutic effectiveness of anti-fungal reagents.

The cells of filamentous fungi grow as tube-like structures comprising multiple cells^25^, and therefore it is difficult to estimate cell number by counting the colony-forming units. Fluorescence imaging allows us to observe and quantify pathogens in animals^34,35^. Infected silkworms were used to evaluate anti-bacterial and anti-fungal drugs by monitoring host survival. By using pathogens expressing eGFP, we were able to observe proliferation of the pathogens in the host as well as suppression of the proliferation by the administration of the anti-fungal drugs against dermatophytosis. This is the first report of the use of fluorescence to detect the growth of pathogens expressing eGFP in silkworms, and as an indicator for evaluating drug efficacy in silkworms.

We demonstrated that germinated conidia had higher pathogenicity than non-germinated conidia in a silkworm dermatophyte infection model. Moreover, mycelial growth of dermatophytes expressing eGFP was observed in silkworm organs, such as the fat body and midgut. Mycelial growth in the silkworms was inhibited by intra-midgut administration of terbinafine. Terbinafine targets squalene epoxidase in dermatophytes and inhibits growth^36^. Phagocytosed dermatophytes elongate their hyphae inside macrophages, leading to rupture of the macrophage membrane *in vitro* ^24^. Based on *ex vivo* observations that dermatophyte hyphae invade the host tissue^22,23^, and *in vitro* observations that phagocytosed dermatophytes elongate their hyphae inside macrophages, leading to rupture of the macrophage membrane^24^, mycelial growth of dermatophytes is considered to be important for dermatophytosis. Our understanding of the molecular mechanisms of mycelial growth *in vivo*, however, is limited. Previous studies demonstrated that the use of silkworm infection models and mutants of pathogens are useful for determining the genes responsible for pathogenicity. In bacteria, virulence genes of *S. aureus, pseudomonas aeruginosa*, and *Bacillus cereus* were identified using silkworms by screening mutants with attenuated lethality in silkworms^37–39^. The gene encoding PTS1 of *C albicans* and the *cyb2* gene of *C. glabrate* were identified as a novel virulence gene or adaptive gene, respectively, in fungi based on screening the fungal mutants using the silkworm infection models^18,19^. Genetic techniques to construct mutant libraries have been established in dermatophytes^28^. Therefore, the silkworm dermatophyte infection model will be useful for determining the factors responsible for the mycelial growth of dermatophytes in animals.

We found that the silkworm was killed by injection of four different species of dermatophytes, including *T. rubrum*, which is the most frequently isolated dermatophyte from patients with dermatophytosis. In a mammalian model using the guinea pig, *T. rubrum* causes less virulence than the other three dermatophytes, and establishing a reproducible system has proved difficult^8^. *T. rubrum* exhibited a similar LD_50_ as *A. vanbreuseghemii* and *M. canis* in the silkworm infection model, suggesting that the silkworm infection model could be useful for screening agents effective against *T. rubrum*.

The silkworm dermatophyte-infection model is useful for evaluating orally administered agents with therapeutic effects. Fluconazole, an orally administered anti-fungal used clinically, had therapeutic effects following midgut injection in the silkworm infection model with *C. neoformans*. On the other hand, midgut injection of amphotericin B, which is not orally available to human patients due to intestinal absorption problems, did not exhibit therapeutic effects in silkworms infected with *C. neoformans* ^12^. Intra-midgut injection in silkworms corresponds with oral administration in humans^12^. In the present study, intra-midgut injection of the anti-fungal medicines terbinafine and itraconazole, which are administered orally to human patients, had therapeutic effects in silkworm. The ED_50_ values of terbinafine and itraconazole were 11 ± 3 and 21 ± 3 mg/kg, respectively, in the silkworm infection model. The recommended daily oral dose of terbinafine and itraconazole for human tinea pedis (dermatophyte foot infection) is 250 mg and 400 mg/person, respectively^40^. Given that the average weight of humans is 60 kg, the calculated values of terbinafine and itraconazole are 4.2 and 6.7 mg/kg, respectively, for humans. Therefore, the therapeutically effective doses of these agents in the silkworm were consistent with the effective doses in humans. The results suggest that the silkworm model is highly valuable for screening orally and therapeutically effective drugs against dermatophytosis.

In conclusion, the silkworm dermatophyte infection model is a quantitative *in vivo* evaluation system for orally administered anti-fungal drugs. Using dermatophytes expressing eGFP allowed us to investigate mycelial growth in the animal. Many active anti-fungal substances have been reported^41–44^. From these candidates, therapeutically effective agents may be discovered using this silkworm model as a second screening tool. This system might contribute to reducing the number of mammalian animals used for discovering novel anti-dermatophyte drugs.

## Methods

### Dermatophytes used in this study

Four species of dermatophytes, *A. vanbreuseghemii*, *A. benhamiae*, *Microsporum canis*, and *T. rubrum*, were used in this study (Table 3). Conidia of dermatophytes were stocked in vials with silica gel at −80 °C.

**Table 3:** Dermatophyte strains used in this study.

Species	Strains	References
*Arthroderma vanbreuseghemii*	TIMM2789	Uchida *et al*.^47^
*Arthroderma benhamiae*	IHEM20161	Symoens *et al*.^48^
*Microsporum canis*	20080	This study
*Trichophyton rubrum*	CBS118892	White *et al*.^49^
*Arthroderma vanbreuseghemii*	AvT-EGFP7	This study

### Culture method

Dermatophyte conidia stocked at −80 °C were spread on modified 1/10 Sabouraud agar (Bacto peptone 0.2%, glucose 0.1%, KH2PO4 0.1%, MgSO_4_ · 7H_2_O 0.1%, Bacto agar 1.5%, pH unadjusted), and incubated for 7–14 days at 28 °C. After incubation, 0.05% Tween80 was added to the plate, and the conidia were collected. The number of conidia was counted using a hemocytometer under a microscope. Conidia were cultured in Sabouraud medium (Bacto peptone1%, glucose 4%) with shaking at 28 °C for 20–28 h and used as germinated conidia.

### Silkworm rearing

Eggs of Fu x Yo x Tukuba ∙ Ne were purchased from Ehime-Sanshu Co., Ltd. (Japan), disinfected, and hatched at 25–27 °C. The silkworms were fed an artificial diet, Silkmate 2 S, containing antibiotics purchased from Nihon-Nosan Co., Ltd. (Japan). Fifth instar larvae were used in the infection experiments.

### Silkworm infection experiments

Silkworm fifth instar larvae were fed 1.5 g artificial diet (without antibiotics, Nihon-Nosan Co., Ltd., Japan) overnight. A suspension (50 μl) of the dermatophyte conidia or germinated conidia was injected into the silkworm hemolymph using a 1-ml tuberculin syringe (Terumo Medical Corporation, USA). To evaluate the therapeutic effects of anti-fungal agents, germinated conidia (4 × 10^6^) were injected into the silkworm hemolymph, and then various concentrations of the anti-fungal agents (50 μl) dissolved in saline were injected into the silkworm hemolymph or midgut. To determine the ED_50_ values, three silkworms were injected for each dose of the anti-fungal agents, and the doses were created by 2-fold serial dilutions.

### Construction of *A. vanbreuseghemii* expressing eGFP

A binary vector, pAg1h-*eGFP*, for the production of *A. vanbreuseghemii* expressing eGFP was constructed as follows. A genomic DNA fragment containing the upstream region (1.9 kb) of the *A. vanbreuseghemii* dipeptidyl peptidase V (*DPPV*) gene was amplified by polymerase chain reaction (PCR) with a pair of primers, AvDPPV-F1 (AAG*ACTAGT*GACAATGACCCACAGGGCAAG) and AvDPPV-R1 (TGC*GGGCCC*TGTGAATGGAGCTAAGTTAATAGC). A DNA fragment containing the *eGFP* gene and the termination sequence of the *Aspergillus nidulans* tryptophan C gene (Accession No. ×02390; *TtrpC*) were also amplified from pCHSH75-GFP/TtrpCby PCR with a pair of primers^28^, eGFP-F1 (CGAAC*GGGCCC*ATGGTGAGCAAGGGCGAGGA) and TtrpC-R1 (AAGCTA*CTCGAG*AAAGAAGGATTACCTCTAA). The two amplified fragments were double-digested with *Spe*I/*Apa*I or *Apa*I/*Xho*I, and inserted into the *Spe*I/*Apa*I and *Apa*I/*Xho*I sites within pAg1-*hph* ^45^, respectively, to generate pAg1h-*eGFP*. The pAg1h-*eGFP* was introduced into the wild-type *A. vanbreuseghemii* strain TIMM2789 using the *Agrobacterium tumefaciens*-mediated transformation method described previously^28^. After co-cultivation, the transformants were screened on Sabouraud agar containing 100–300 μg/ml hygromycin B. The desired transformants were selected by Southern blotting analysis and fluorescence microscopic observation. The transformant AvT-EGFP7 used in this study was confirmed to harbor two copies of the *eGFP* gene in the chromosomes.

### Imaging of mycelial growth in silkworms

Conidia of *A. vanbreuseghemii* expressing eGFP were injected into silkworms, followed by injection with terbinafine (20 μg in saline) into the silkworm midgut. The silkworms were reared at 30 °C. Fat bodies and midguts were isolated after 1, 2, and 3 days. Small pieces of isolated tissues were placed on glass slides and pressed flat by cover slips^46^. The samples were examined under a microscope with bright light or ultraviolet light using a microscope equipped with fluorescent lens (Olympus). For quantitative evaluation of the fluorescence, fat body tissues were homogenized by sonication and centrifuged pellets (13 krpm, 3 min) were suspended in 10 μl of water. Suspended samples (0.5 μl) were dropped on a microscope slide, dried, and photographed under a fluorescence microscope. Fluorescence of the images was determined by Image J software (ImageJ 1.47t; National Institutes of Health, Bethesda, MD).

### MIC determination

The MIC was determined according to the CLSI M38-A2 method. Conidia (2 × 10^3^) were incubated with 2-fold serial dilutions of anti-fungal agents at 35 °C for 4 days, and MIC_80_ (concentration required to inhibit growth by 80%) was determined.

### Anti-fungal agents

Terbinafine was purchased from Wako Pure Chemical Industries, Ltd. (Osaka, Japan). Itraconazole was obtained from Sigma-Aldrich Corporation (St. Louis, MO, USA). The compounds were dissolved in dimethyl sulfoxide at a concentration of 10 mg/ml as stock solution. The stock solution was diluted with saline for therapeutic assay using the silkworms.

### Statistical analysis

Survival curves were determined for each group based on the log-rank test, and p-values were determined. The log-rank test was performed for statistical processing using Prism software (GraphPad Software, Inc.).
